# Human immunodeficiency virus 1 glycoprotein 120 induces endoplasmic reticulum stress in neurons

**DOI:** 10.1038/s41419-025-08032-x

**Published:** 2025-10-06

**Authors:** Christy Agbey, Lee A. Campbell, Thieu Phan, Gerard Ahern, Italo Mocchetti

**Affiliations:** 1 Interdisciplinary Program in Neuroscience, Washington, DC USA; 2 Department of Neuroscience, Georgetown University Medical Center, Washington, DC USA; 3 Department of Pharmacology, Georgetown University Medical Center, Washington, DC USA

**Keywords:** Pathogenesis, Neuroscience, Mechanisms of disease

## Abstract

People living with Human Immunodeficiency Virus (HIV) (PLWH) may develop HIV-associated neurocognitive disorder (HAND) despite the use of antiretroviral therapy. Therefore, more studies are needed to identify novel therapies, which require a better understanding of the molecular and cellular mechanisms underlying HIV neurotoxicity. The HIV envelope protein gp120 causes neuronal degeneration similar to that observed in HAND. One mechanism contributing to gp120-mediated neurotoxicity may involve its ability to inhibit protein processing in the Golgi apparatus and endoplasmic reticulum (ER). To provide data in support to this hypothesis, we have used a variety of experimental approaches to investigate the effect of gp120 on ER dynamics. We first analyzed the levels of ER stress-associated proteins, such as immunoglobulin heavy chain binding protein (BiP) and phosphorylated Inositol-Requiring Enzyme 1 alpha (p-IRE1α) by western blot, as well as ER morphology by electron microscopy in gp120 transgenic (tg) mice. We found that the hippocampus of gp120tg mice exhibits an increase of BiP levels and p-IRE1α, as well as altered ER morphology when compared to wild type mice. We confirmed that gp120 alters ER morphology in neurons by using rat cortical neurons in culture. The effect of gp120 was chemokine-co-receptor dependent because AMD3100, a CXCR4 receptor antagonist, abolished the effect of gp120 on BiP immunoreactivity. Moreover, using Gluc-ASARTDL, a reporter protein for monitoring ER calcium, and live Ca^2+^ imaging, we show that gp120 induces ER Ca^2+^ depletion in neurons. Overall, our data suggest that gp120 promotes ER stress in neurons.

## Introduction

Up to 50% of people living with human immunodeficiency virus-1 (HIV) (PLWH) may develop a spectrum of neurocognitive impairments, termed HIV-associated neurocognitive disorder (HAND) [1]. Symptoms of HAND include memory and learning impairments, executive dysfunction, motor difficulties, mood and behavioral changes, and more [2, 3]. Despite the effectiveness of combination antiretroviral therapy (ART) in reducing mortality rates and delaying the progression to AIDS among PLWH, the incidence of HAND remains unchanged [4]. This suggests that ART may not effectively eradicate HIV within the central nervous system (CNS), and it underscores the need to uncover the pathology underlying HAND in order to achieve improved therapeutic outcomes.

The neuropathology of HAND includes neuroinflammation, neuronal loss, toxic protein accumulation, and more [5, 6]. However, the mechanisms underlying the neuropathologic features seen in HAND are not entirely clear. HIV enters the CNS shortly after peripheral infection. In the brain, HIV does not productively infect neurons; however, evidence suggests that specific viral proteins released by HIV-infected cells contribute directly to the observed neurodegeneration. At least four viral proteins have been shown to induce neurotoxicity by contributing to chronic inflammation and promoting neuronal degeneration [7–10]. One of the most intriguingly studied viral proteins implicated in HAND pathology is gp120, the HIV envelope glycoprotein, which induces neuronal degeneration both in vivo [11] and in vitro [12–16]. Gp120 expression has been detected in the cerebrospinal fluid of PLWH treated with ART [17], demonstrating its continued relevance to HAND despite effective ART. The neurotoxic mechanism of gp120 initiates after its binding to the neuronal chemokine co-receptors, CCR5 and CXCR4 [13, 15], followed by its internalization into neurons [18, 19] and trafficking to the endoplasmic reticulum (ER) [20]. Once inside neurons, gp120 induces several neurotoxic mechanisms, including the reduction of axonal transport [16], mitochondrial pathology [21], and the inhibition of endoproteases located in the ER [14]. This suggests that gp120 neurotoxicity include mechanisms that alter the ER or related structures within the ER, which, in turn, may affect ER dynamics.

The ER is a cellular organelle that aids in maintaining cellular homeostasis by serving as a site for protein synthesis and calcium storage [22]. Under pathologic conditions, the ER may become stressed. The ER stress response may be triggered by misfolded or aggregated protein, as well as by dysregulated calcium dynamics [23]. ER stress is sensed by a network of proteins referred to as the unfolded protein response (UPR), which involves the activation of signal transduction pathways that promote either cell death or cell survival. Prolonged or severe ER stress evokes neuronal dysfunction [24]. ER stress and UPR upregulation have been implicated in the pathogenesis of a number of neurodegenerative diseases, including Alzheimer’s and Parkinson’s disease [25, 26]. The underlying HAND pathology suggests that ER stress may also be a contributing factor in disease progression, though its role in HAND remains under-investigated. Additionally, postmortem studies have found that the brains of PLWH exhibit increased deposition of amyloid-beta [27**–**29], a protein that is generated, processed, and trafficked through the ER-trans-Golgi network. This suggests that protein misfolding, often linked to ER disruption, may contribute to HAND.

In this paper, we investigated the effect of gp120 on the ER to better elucidate the mechanisms underlying HAND pathology using both in vitro and in vivo approaches. We present data that gp120 modifies ER morphology and function, suggesting that gp120 induces ER stress and alters ER dynamics.

## Materials and methods

### Animals

Gp120 transgenic (gp120tg) mice on the C57BL/6 N genetic background were obtained from the original colony [11] and bred in our vivarium. Wildtype (WT) mice were generated from this colony and used as controls. In total, we used 12 WT and 12 gp120tg mice in this study, (*n* = 9 per group for western blot analyses, and *n* = 3 per group for electron microscopy), and males and females were balanced among our experimental groups. Based on effect sizes, *n* = 22 mice were determined using power analysis (alpha = 0.05, power = 0.8) prior to beginning experimentation. 2–4 mouse littermates were housed per cage in the Georgetown University Department of Comparative Medicine. Mice were kept on a 12 h light-dark cycle with food and water *at libitum*, and were entered into the study at 52–66 weeks and randomly assigned to experimental groups.

### Ethics approval

Experiments involving animals were approved by the Institutional Animal Care and Use Committee at Georgetown University (protocol number 2016-1188).

### Euthanasia and tissue processing

At 52–74 weeks, animals were anesthetized with Ketamine/Xylazine (80/10 mg/kg, i.p.). Mice were perfused through a needle inserted into the left ventricle with 10–20 ml of ice-cold phosphate buffered saline (PBS). The whole brains were extracted, chilled in PBS, and micro-dissected using a 1 mm brain block (cat. no. RBMS-200C, Kent Scientific, Torrington, CT, USA). The extracted neural tissue was immediately frozen and stored at −80 °C.

### Rat cortical neurons

Primary rat cortex neurons (RCN) were purchased from ThermoFisher Scientific, Waltham, MA (cat. no. A10840-01). RCN were plated ~1 × 10^5^ live cells/well in Poly-D-Lysine (cat. no. A3890401, ThermoFisher Scientific) coated 24-well plates (cat.no. 3526, Corning, NY, USA) on 12 mm diameter coverslips (#1 thickness) (cat. no. 50-121-5159, ThermoFisher Scientific), and incubated at 36–38 °C in a humidified atmosphere of 5% CO_2_ in air. RCN were fed every third day by aspirating half of the medium from each well and replacing it with fresh warmed medium.

On day-in-vitro 14 (DIV 14), RCN were exposed to either vehicle control (VEH), consisting of 0.1% bovine serum albumin (BSA), or 5 nM recombinant gp120IIIB (cat. no. 1001, ImmunoDx, Woburn, MA, USA) dissolved in 0.1% BSA. RCN were also exposed to gp120 following a 15 min pretreatment with 5 μM AMD3100 (cat. no. A5602, Sigma-Aldrich, St. Louis, MO, USA). RCN were also exposed to either nef or tat (cat. no. ARP-11478 and ARP-2222, National Institute of Health, HIV Reagent program, Bethesda, MD, USA), or thapsigargin (cat. no. T9033, Sigma-Aldrich).

### Immunocytochemistry

Twenty four hours post-exposure to the above treatments, RCN were fixed in 4% paraformaldehyde (PFA) for 20 min, blocked for 30 min in 5% PBS-X, then incubated with anti-GRP78/BiP (1:500, cat. no. ab21685, Abcam, Cambridge, UK) antibody overnight. The following day, cells were incubated with Alexa Fluor-488 goat anti-rabbit secondary (1:2000, cat.no. A11008, ThermoFisher Scientific) for 1 h. 4′,6-diamidino-2-phenylindole (DAPI) (1:10,000, cat. no. D9542, Sigma-Aldrich) was used as a nuclear marker. Images were taken in three randomly selected fields of view/well via an OlympusIX71 microscope (Breinigsville, PA). Three biological replicates from different cultures were performed, with at least two technical replicates each.

### Western blot

Both RCN lysates and hippocampal tissue homogenates of 12–17-month-old WT and gp120tg mice were prepared in a mixture of RIPA buffer (cat. no. 20-188, 124 Sigma-Aldrich) containing protease-phosphatase inhibitors (cat. no. 78442, ThermoFisher Scientific). Protein content was determined via a BCA Protein Assay Kit (cat.no. 23225, ThermoFisher Scientific). Proteins (15 µg/sample) were separated on NuPAGE 4–12% Bis-Tris Gels (cat. no. NP0335, ThermoFisher Scientific) and transferred to a 0.2 µm PVDF membrane (cat. no. 1620117, Bio-Rad Laboratories, Hercules, CA, USA). Membranes were blocked in 5% milk in PBS and 0.1% Tween-20, followed by overnight incubation with anti-GRP78/BiP antibody (1:1000, cat. no. ab21685, Abcam), anti-phospho-IRE1α (1:1000, cat. no. PA1-16927, ThermoFisher Scientific), or anti-CHOP (1:1000, cat. no. MA1-250, ThermoFisher Scientific). The following day, membranes were incubated for 1 h in one of the following corresponding secondary antibodies: goat anti-rabbit IgG HRP (1:2000, cat. no. 31463, ThermoFisher Scientific) or goat anti-mouse IgG HRP (1:2000, cat. no. 31430, ThermoFisher Scientific). Blots were imaged using Pierce ECL Western Blotting Substrate (cat. no. 32209, ThermoFisher Scientific). Restore Western Blot Stripping Buffer (cat. no. 21059, ThermoFisher Scientific) was applied for 15 min before re-probing with anti-β-actin antibody (1:5000, cat. no. A1978, Sigma-Aldrich) as a loading control. The experiment was replicated three times, with *n* = 3 animals per experimental group each time.

### SH-SY5Y Gluc-ASARTDL cells

SH-SY5Y human neuroblastoma (cat. no. CRL-2266, ATCC, Manassas, VA, RRID:CVCL_0019) were grown as previously described [30] with minor modifications. Cells were transduced with a lentivirus to express *Guassia* luciferase (Gluc)-ASARTDL and maintained by an established protocol [31]. At 80% confluence, cells were plated at ~2.8 × 10^5^ live cells/mL into 96-well Nunclon Delta-Treated microplates (cat. no. 136101, ThermoFisher Scientific). Exposure to drugs was performed 24 h after plating. Luciferase was measured in the medium by adding coelenterazine (CTZ) (cat. no. C2230, Sigma-Aldrich) and using a SpectraMax iD3 plate reader (Molecular Devices) and the SoftMax Pro software. Three independent experiments were performed, with two technical replicates each time.

### Transmission electron microscopy

Hippocampal tissue from 12-month-old wildtype and gp120tg mice (*n* = 3, each) was cut into 1–2 mm thick slices and fixed in 2.5% glutaraldehyde fixative (pH 7.0), starting at room temperature and after 15–30 min continued at 4 °C. Samples were sent to Creative Bioarray (Shirley, NY, USA), where transmission electron microscopy (TEM) was performed with a Hitachi HT7700 TEM operating microscope.

### Calcium imaging

On DIV14, RCN were exposed to either vehicle or 5 nM gp120IIIB, as detailed above. Twenty four hours post-exposure, we performed calcium imaging using a fluorescent Ca^2+^ indicator (Fluo-4 AM, 4 μM, ThermoFisher Scientific) in physiologic buffer (140 mM NaCl, 4 mM KCl, 10 mM HEPES, 5 mM glucose, 2 mM CaCl_2_, and 1 mM MgCl_2_ at pH 7.3). The dye was excited at 480 ± 15 nM. Emitted fluorescence was filtered with a 535 ± 25 bandpass filter. Real-time imaging was conducted using a Qimaging camera (Retiga 3000 M) and read onto a computer with Mosaic V2 Image Capture and Analysis Software (Tucsen, Fujian, China). Analysis was performed using ImageJ and the Time Series Analyzer v3 plugin to generate values for change in fluorescence above baseline (ΔF). The experiment was repeated twice with two different neuronal cultures with at least two technical replicates each time.

### Statistical analysis

For western blot, the relative density of bands was performed using ImageJ. Data were collected and organized in Microsoft Excel. Normalization was performed for calcium imaging by dividing the fluorescence value for a cell at a given time point by the baseline value (*F/F*_*0*_), and for SERCaMP by dividing luminescence values at different concentrations by the baseline value. The generation of graphs and all statistical measures were performed with GraphPad Prism v.7.0 (Graphpad Software Inc., San Diego, CA, USA). Data are expressed as the mean ± SD, and analyzed with an unpaired *t*-test or one way ANOVA and Tukey’s post-hoc. A *p* < 0.05 was considered statistically significant and normal distribution and equal variance were assumed for all statistical testing. Experimental groups were randomly assigned by number and researchers were blinded during experimentation and analysis for all cell treatments, calcium imaging, and immunocytochemistry (ICC). For in vitro experiments, a biological replicate represents an independently prepared neuronal culture, treated in separate plates and cultured on a different day. A technical replicate refers to multiple wells or measurements taken from the same culture.

### Illustrations

Figures were created within Inkscape and ZEN (Zeiss, Oberkochen, Germany). Graphics were created within BioRender (Toronto, ON).

## Results

### Increased expression of ER stress marker BiP in gp120tg mice

**Fig. 1:**
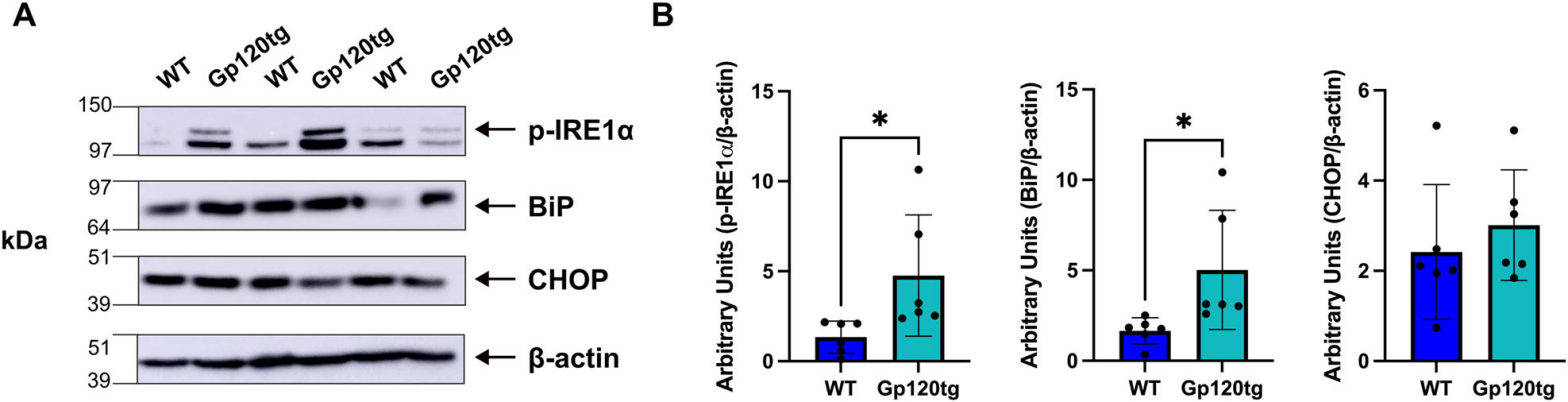
The expression of ER stress-associated proteins is increased in the hippocampus of aged gp120 transgenic mice. **A** Example of western blot analysis of lysates prepared from the hippocampus of the indicated mouse genotypes. Blots were probed with antibodies against BiP, p-IRE1α, or C/EBP homologous protein (CHOP). β-actin was used as a loading control. Molecular weights in kDa of analyzed proteins are listed on the left. BiP expression was detected at around the expected 78 kDa molecular weight, and p-IRE1α at around 110 kDa **B** Levels of proteins, expressed as arbitrary units, were quantified by dividing the intensity of BiP, p-IRE1α, or CHOP band to the corresponding β-actin band. Data are expressed as mean ± SD. *p = 0.0494 and *p = 0.0344 for p-IRE1α and BiP respectively; two-tailed unpaired *t*-test. *N* = 6 animals per experimental group. Blinding was not possible.

ER stress occurs when misfolded proteins accumulate in response to stressful stimuli, triggering an adaptive response by the ER to counteract the stressful conditions and restore homeostasis. To investigate whether gp120 induces ER stress, we first investigated the levels of immunoglobulin heavy chain binding protein BiP, an ER chaperone protein that may act as an ER stress sensor, and phosphorylated Inositol-Requiring Enzyme 1 alpha (p-IRE1α), a key UPR protein [32]. We prepared brain homogenates from the hippocampus of 12–17-month-old gp120tg and WT mice. Gp120tg mice manifest key neuropathological features of HAND such as decreased dendritic spines, synaptic simplification, and cognitive and motor deficits demonstrated by performance in behavioral assessments, including the Morris water maze and Barnes maze, open field, light/dark transition task, and prepulse inhibition tests [11, 33–35]. Western blot analysis revealed that the levels of ER-stress associated proteins BiP and p-IRE1α are increased in the hippocampus of gp120tg mice when compared to WT (Fig. 1). Full length original blots are included as supplemental material.

### Gp120 induces ER stress morphology

**Fig. 2:**
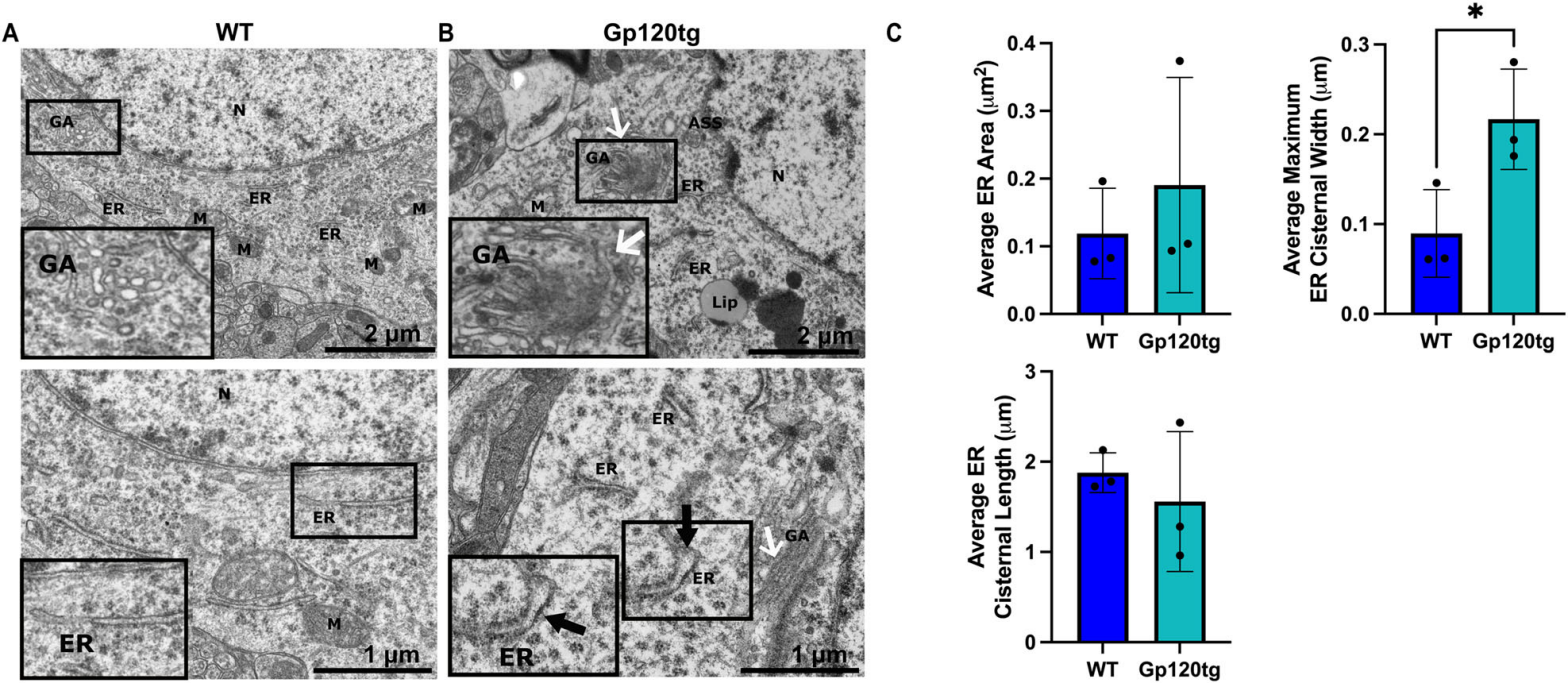
ER morphology is altered in gp120tg mice. Representative images of transmission electron microscopy analysis of two random fields within the hippocampus of **A** WT and **B** gp120tg mice. Images were acquired with Hitachi HT7700 TEM operating microscope. Black arrows point to ER stress morphology, including enlarged ER lumen. White arrows point to GA displaying pathologic morphology characterized by enlarged and disorganized cisternae and vesicles. Images show stress-associated ER and GA morphology in the gp120 transgenic mouse hippocampus. Corresponding images in each panel show different magnifications. Bar = 2 μm, and 1 μm. **C**. To quantify ER morphology, we measured the ER cisternal area, the maximum cisternal width across the widest luminal diameter, and the length of continuous ER cisternae. Each data point represents the average of 3–4 ER cisternae measured from a single electron micrograph per mouse (*n* = 3 mice per group). Data is expressed as mean ± SD. *p = 0.0412; two-tailed unpaired *t*-test. ASS autolysosome, Lip lipofuscin, M mitochondria, N nucleus.

The accumulation of proteins in the ER in response to stress may induce changes in ER structure. To determine whether gp120 induces ER stress associated morphology, we used TEM to visualize the ER and other closely related structures on a subcellular level in the hippocampus of 12-month-old gp120tg and WT mice (Fig. 2). The ER lumen of gp120tg mice appears to be enlarged (Fig. 2B) when compared to that of WT mice (Fig. 2A). Quantitative morphometric analysis confirmed these findings, with significantly increased maximum ER cisternal width, representative of ER lumen swelling, in the gp120tg mice (Fig. 2C). In addition, structures closely related to the ER appear to be affected. Specifically, the Golgi apparatus (GA), an organelle involved in the trans-ER-Golgi-transport system, exhibits morphological pathology. This includes a lack of organized cisternae, fragmentation into small vesicles, and abnormal clustering (Fig. 2B). The degree of GA disorganization in the gp120tg mice precluded consistent morphometric quantification. The structural differences were exceptionally notable when compared to the morphology of the ER and GA in the WT mice (Fig. 2A). WT mice exhibit healthy ER morphology, which appear as flattened tubular structures, and healthy GA morphology, with organized cisternae and vesicles (Fig. 2A).

### Gp120, ER morphology, and chemokine-coreceptor

**Fig. 3:**
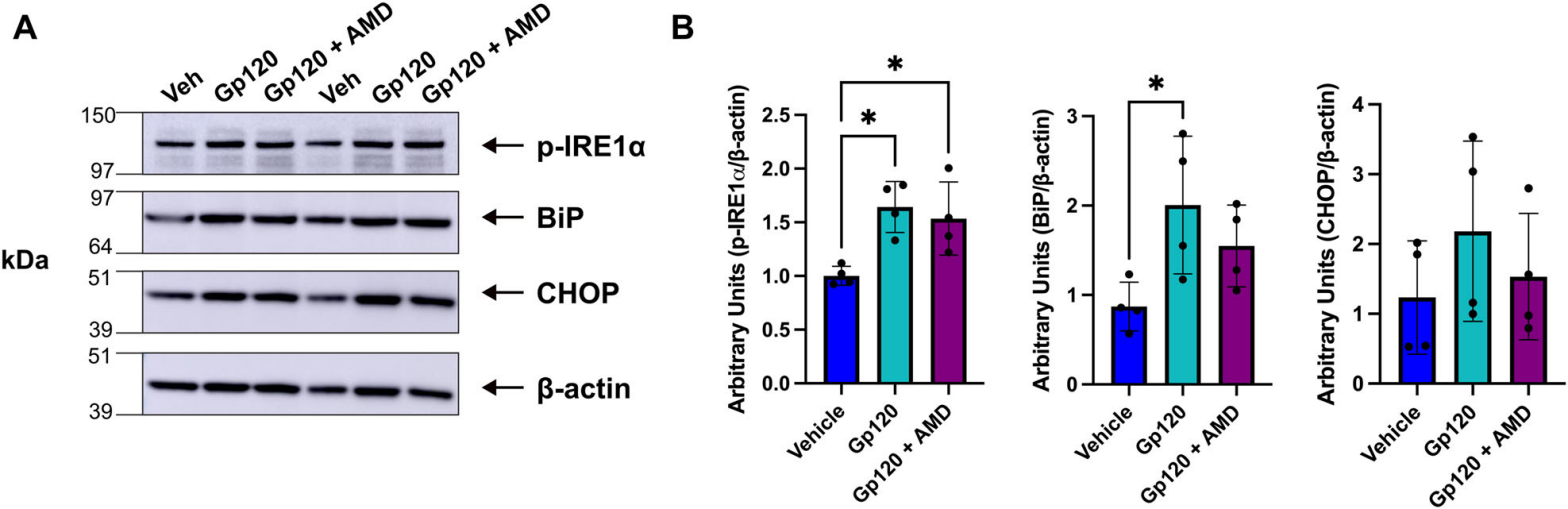
The expression of ER stress-associated proteins is increased in gp120-exposed primary neuronal cultures. **A** Example of western blot analysis of lysates prepared from RCN exposed to either vehicle control (0.1% BSA), gp120 IIIB, or gp120 IIIB following pretreatment with AMD3100. Blots were probed with antibodies against BiP, p-IRE1α, or CHOP. β-actin was used as a loading control. Molecular weights in kDa of analyzed proteins are listed on the left. **B** Levels of proteins, expressed as arbitrary units, were quantified by dividing the intensity of the BiP, p-IRE1α, or CHOP bands to the corresponding β-actin band. Data are expressed as mean ± SD. *p = 0.0107 and *p = 0.0451 vehicle vs gp120 for p-IRE1α and BiP, respectively; one-way ANOVA with Tukey’s post-hoc. *N* = 4 lysates from different cultures per experimental group. Blinding was not possible.

**Fig. 4:**
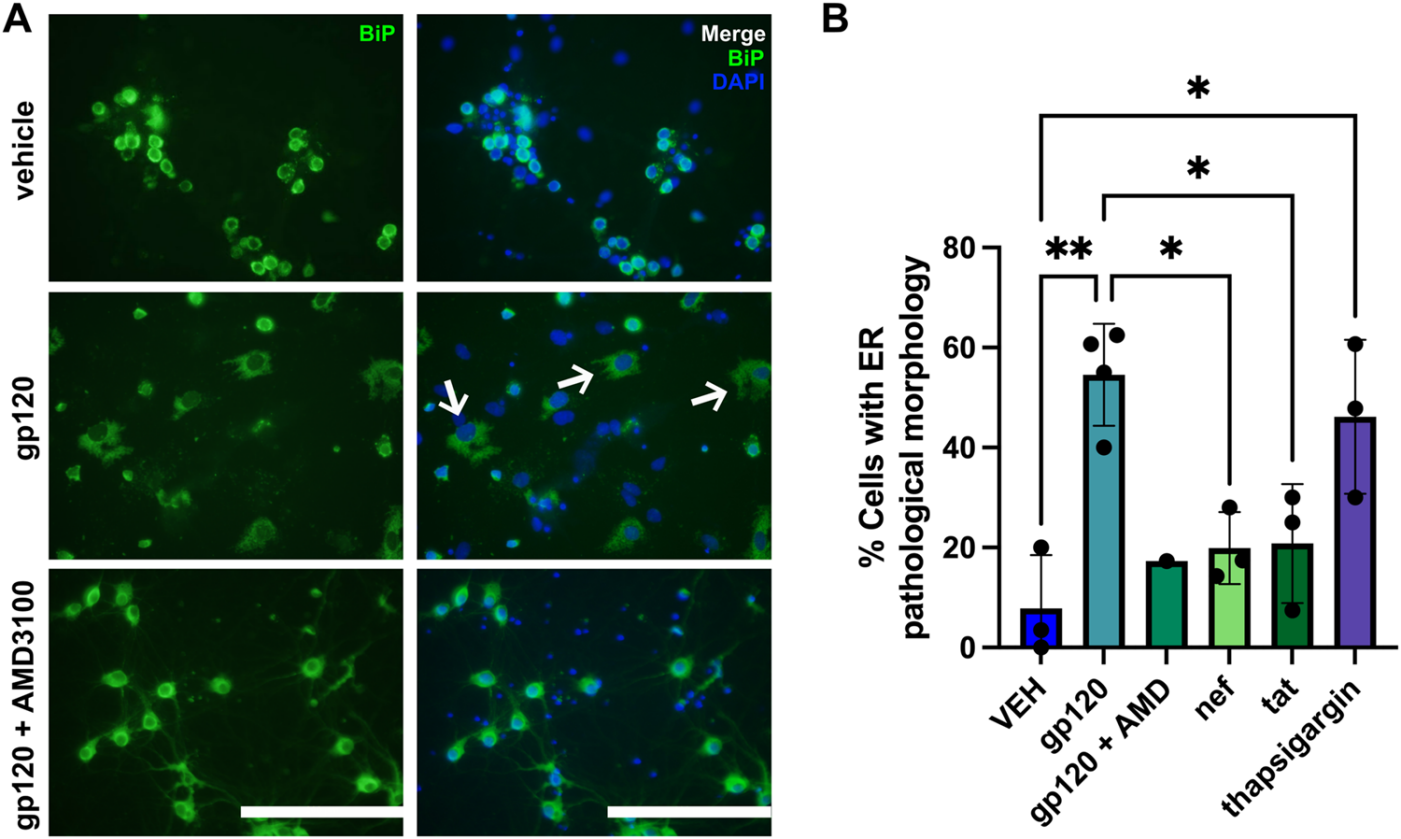
Gp120 induces ER stress morphology in rat cortical neurons. **A** Representative images of RCN. On 14 days in vitro, neurons were exposed to vehicle control, 5 nM gp120IIIB alone, 5 nM gp120IIIB following a 15-min pretreatment with AMD3100, the HIV proteins nef or tat, or thapsigargin. Twenty four hours later, cells were fixed in 4% PFA and ER morphology was observed by immunocytochemistry using an anti-BiP antibody (green). DAPI (blue) was used to stain nuclei. ER stress-associated morphology, including enlarged lumen with ER membrane, is observed in neurons exposed to gp120, but not in vehicle exposed neurons or in neurons pretreated with AMD3100. White arrows point to representative neurons exhibiting the described ER stress morphology. Bar = 200 μm. Images were acquired on an Olympus IX71 fluorescence microscope and images were taken in three randomly selected fields of view per well. **B** The % cells that exhibit the described ER morphological pathology were quantified manually using ImageJ. An average of 54% of cells exposed to gp120 and 46.2% of cells exposed to thapsigargin exhibited ER stress morphology, which were significantly higher than the percentage of cells exposed to vehicle (7.6%) that exhibit the described stress-associated morphology (**p = 0.0022 VEH vs gp120 and *p = 0.0150 VEH vs thapsigargin; One-way ANOVA with 26,574 Tukey’s post-hoc). Pretreating cells with AMD3100 prior to gp120 exposure abolished the effect of gp120 on ER morphology, with an average of 17.3% of cells exhibiting abnormal ER morphology. An average of 19.9% of cells exposed to nef and 20.8% of cells exposed to tat exhibited abnormal ER morphology, which were not significantly different to vehicle-exposed cells. Data are expressed as mean ± SD and analyzed with one-way ANOVA with Tukey’s post-hoc. The experiment was repeated three times with three different cultures of neurons (three biological replicates). Experimental groups were randomly assigned and researchers were blinded during experimentation and analysis.

To determine whether the effect of gp120 occurs in neurons and is through the chemokine-coreceptor CXCR4, we utilized RCN. RCN develop in vitro to resemble mature neurons morphologically and physiologically, and are equally sensitive to the neurotoxic action of gp120 when compared to neurons prepared from other brain areas [12, 13, 36]. Thus, these neurons were used as a representative in vitro model to investigate neuronal mechanisms, without the intent to draw region-specific conclusions. We exposed RCN to either a vehicle (VEH) control (0.1% BSA) or 5 nM gp120IIIB, alone or in combination with AMD3100, a CXCR4 receptor antagonist [37] (Fig. 3). Twenty four hours post-exposure, cell lysates were collected for western blot, or cells were fixed in 4% PFA for ICC. Western blot analysis revealed that RCN exposed to gp120 exhibited increased expression of the ER stress markers BiP and p-IRE1α (Fig. 3). Pretreatment of RCN with AMD3100 blocked the effect of gp120 (Fig. 3). Via ICC, we found that exposure of RCN to gp120 for 24 h resulted in abnormal ER morphology characterized by an enlarged ER lumen (Fig. 4A). The percentage of gp120-exposed cells which exhibited this morphology varied between 40% and 63% in a given field of view. In contrast, most vehicle-exposed neurons exhibited healthy ER lumens, with only an average of 7.6% of cells exposed to VEH exhibiting abnormal ER morphology in a given field of view. To thoroughly validate that any observed pathology is specific to gp120, cells were also exposed to 100 nM of the HIV proteins nef or Tat. Additionally, RCN were exposed to thapsigargin, which induces ER stress by inhibiting the sarco/ER Ca²⁺ ATPase (SERCA) and depleting ER calcium [38]. Thapsigargin promoted a pathology with ER enlargement, similar to gp120 (Fig. 4B), with an average of 46.2% of cells within a given field of view exhibiting this abnormal morphology. Pretreatment of cells with AMD3100 prior to gp120 exposure, abolished the toxic effect of gp120 on the ER (Fig. 4). Exposure of RCN to nef and tat did not lead to abnormal ER morphology (Fig. 4B). Representative images of neurons exposed to tat, nef, or thapsigargin are shown in Supplementary Fig. 1. Thus, the effect of gp120 on the ER appears to be selective.

### Gp120 induces ER calcium depletion

**Fig. 5:**
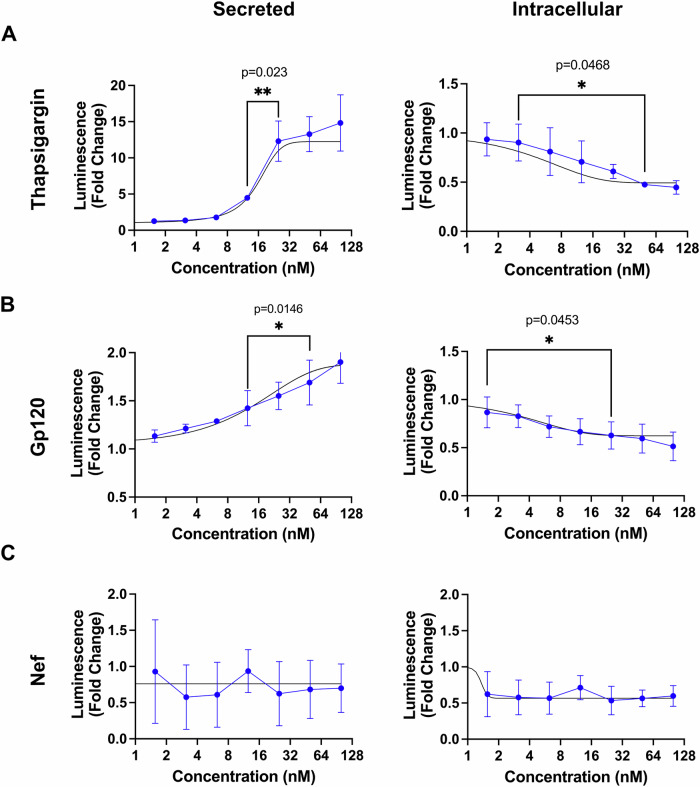
SH-SY5Y cell exposure to gp120 induces ER calcium depletion. SH-SY5Y cells were transduced with an adeno-associated virus to express Gluc-ASARTDL. Transduced cells were exposed to thapsigargin **A**, gp120 **B**, or Nef **C** for 24 h. Luciferase activity was then measured in the medium (secreted) or cell lysates (intracellular) using a plate reader. Data are expressed as the fold change over baseline luminescence. *p < 0.05, **p < 0.01; One way ANOVA, Tukey post hoc. Experimental groups were randomly assigned. The experiment was repeated three times independently with two technical replicates each time.

A major role of the ER is to serve as site for calcium storage, and the disruption of ER calcium homeostasis is a well-known inducer and consequence of ER stress [24, 39]. To assess the effect of gp120 on ER calcium dynamics, we utilized a luciferase-based secreted ER calcium monitoring protein (SERCaMP) assay, in which SH-SY5Y human neuroblastoma cells were transduced with a lentivirus expressing Gluc-ASARTDL [31]. Gluc is a *Gaussia* luciferase, an oxidative enzyme that produces bioluminescence, which can be measured. ASARTDL (alanine-serine-alanine-arginine-threonine-aspartic acid-leucine) is a carboxy terminal peptide amino acid sequence that is specific to the ER-resident protein mesencephalic astrocyte-derived neurotrophic factor (MANF). MANF is localized to the lumen of the ER and is secreted in response to ER calcium depletion and ER stress. Cells were exposed to gp120 in 2-fold serial dilutions (100 nM at the highest). As a positive control, cells were exposed to thapsigargin. To confirm that results are specific to gp120, cells were also exposed to the HIV protein nef. Both thapsigargin and gp120 promoted a concentration-dependent Ca^2+^ efflux from the ER, as evidenced by increased luminescence in the culture medium and a decrease in luminescence intracellularly (Fig. 5A, B). This effect was not observed with exposure to the HIV protein nef, as we did not see a significant change in luminescence extracellularly or intracellularly (Fig. 5C).

### Gp120 and intracellular Ca^2+^

**Fig. 6:**
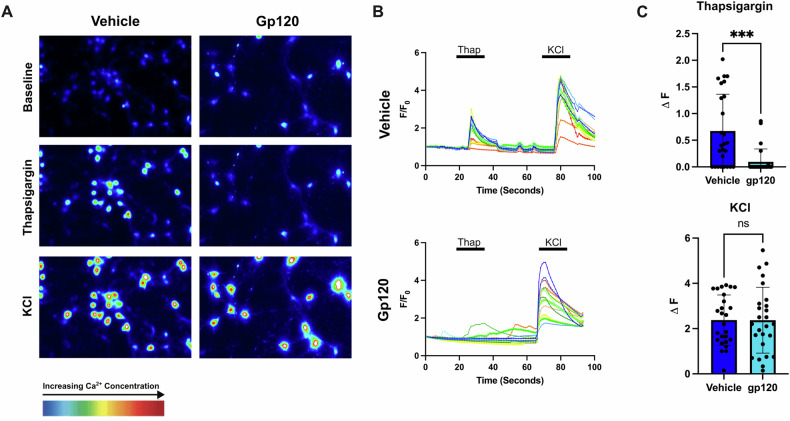
Gp120 induces ER calcium depletion in rat cortical neurons. Primary cortical neurons were exposed to either vehicle control or 5 nM gp120IIIB at DIV 14. Twenty four hours later, Ca^2+^ imaging was performed, during which neurons were imaged at baseline, after the addition of 500 nM thapsigargin, and after the addition of 40 mM KCl. **A** Representative Fluo-4 fluorescence images of cortical neurons captured before (baseline) and after application of thapsigargin and then KCL. **B** Representative average intensity, or mean Fluo-4 fluorescence, for a cell depicting the steep Ca^2+^ concentration increase in the cell as a result of the addition of thapsigargin at ~30 s, and KCl at ~70 s. **C** ΔF, or change in fluorescence, in response to thapsigargin or KCl was measured for each cell. Data are expressed as mean ± SD. **p < 0.0002; two-tailed unpaired *t*-test. *n* = 26 cells/experimental group. The experiment was repeated twice independently (two biological replicates) with at least two technical replicates each time, during which researchers were blinded to the experimental groups and neurons were randomly assigned to the experimental groups. Neurons were randomly chosen for experimental analysis, in which every third or fourth neuron in a given well was analyzed.

To further confirm the effect of gp120 on ER calcium, we utilized Ca^2+^ imaging in live neurons. RCN were exposed to either VEH or gp120IIIB for 24 h prior to Ca^2+^ imaging. For Ca^2+^ imaging, we began imaging cells at baseline and then applied thapsigargin, which resulted in an acute, predictable efflux of Ca^2+^ from the ER in vehicle-exposed neurons (Fig. 6A). About 60–90 s later, we applied KCl, which depolarizes the cell membrane leading to the opening of voltage gated calcium channels. Twenty-six random cells per treatment group were analyzed, and the mean fluorescence intensity (F/F0) over time for each cell was calculated. Cells exposed to vehicle exhibited two peaks in fluorescence intensity, the first representing an increase in Ca^2+^ concentration [Ca^2+^] due to the addition of thapsigargin, and the second representing the increase in [Ca^2+^] due to KCl (Fig. 6B). In contrast to the control, many gp120-exposed RCN did not respond to thapsigargin, evidenced by only one prominent peak in mean fluorescence intensity in response to KCl (Fig. 6B). We next analyzed the change in fluorescence (ΔF) for individual cells in response to thapsigargin or KCl (Fig. 6C). Indeed, we observed that most cells exposed to gp120 for 24 h either did not respond to thapsigargin or exhibited a small response (Fig. 6C). The response of these cells to KCl was unaffected (Fig. 6C), supporting the suggestion that gp120 acts on the ER Ca^2+^ store.

## Discussion

We investigated neuronal ER stress as a mechanism of HIV-mediated neurodegeneration. While gp120-induced ER stress has been reported in other cell types, whether ER stress occurs in neurons, cells which are not productively infected by HIV, is understudied. Our data show that several measures of ER stress are affected by the viral protein gp120. These include an increase in the expression of the ER stress-related proteins BiP and p-IRE1α. In addition, gp120 elicits an enlarged ER lumen both in vivo and in vitro, and depletion of in [Ca^2+^] in cortical neurons. Our data indicate that gp120 promotes ER stress in neurons, highlighting a neuron-specific mechanism of injury relevant to HAND.

We show that BiP and p-IRE1α expression is upregulated in gp120tg mice and gp120-exposed RCN. BiP is an ER chaperone protein [32] that assists in the folding of proteins, targets misfolded proteins for degradation [23], and helps regulate calcium homeostasis [39]. BiP plays important roles in the translocation of proteins that are synthesized in the ER, and in sensing ER stress. In fact, BiP has been referred to as a “master regulator of ER stress” [40]. Under conditions in which the ER is stressed, BiP expression is upregulated. IRE1α is also a critical component of the UPR. When ER stress occurs, IRE1α is phosphorylated, leading to its activation and the initiation of the IRE1α arm of the UPR, which aims to enhance ER protein-folding capacity and remove misfolded proteins through degradation [41]. While adaptive in the short term, prolonged ER stress leads to a dysregulation in ER function and, ultimately, apoptosis. Thus, prolonged UPR activation is a possible mechanism explaining gp120-mediated neurotoxicity.

While the functional neuronal outcomes associated with gp120, including neuronal apoptosis and synaptic loss, have been extensively characterized [12, 13, 15], the mechanisms underlying such outcomes remain unclear. We show that neurons exposed to gp120 IIIB, a X4 preferred ligand, exhibit increases in markers of ER stress and display a prominently enlarged ER lumen when stained with an antibody against BiP, which is present in the lumen of the ER. These morphological characteristics were not observed in neurons pretreated with AMD3100, a CXCR4 antagonist that prevents gp120 IIIB endocytosis and neurotoxicity [19, 37, 42]. In addition, other HIV proteins, such as nef and Tat, did not alter ER morphology, suggesting that these effects are specific to gp120 and require the CXCR4 co-receptor. Furthermore, we observed by electron microscopy that the ER appears to be enlarged in the hippocampus of gp120tg mice when compared to that of WT mice. ER structure consists of a network of tubules, flattened sacs, and sheets. When ER stress is prolonged or severe, the ER lumen may appear enlarged due to the accumulation of misfolded proteins within the ER, a phenomenon that we observed in response to gp120. We also observe that closely related structures, specifically the GA, exhibit pathologic morphology characterized by disorganized cisternae and vesicles. The GA and ER interact to ensure the proper modification, processing, and trafficking of proteins, lipids, and vesicles. ER stress can indirectly impact the GA by causing chronic inflammation and reducing the trafficking of properly folded proteins to the GA. Likewise, dysfunction in the GA may affect protein transport through the trans-ER-Golgi network and lead to ER stress. This might explain our previous data showing that in both gp120 animals and HAND brains, the levels and activity of furin, an endoprotease located in the GA, are downregulated [14]. Importantly, reduced levels of furin lead to accumulation of neurotoxic protein such as pro-brain derived neurotrophic factor [14], which promotes neuronal degeneration [43]. Thus, ER dysfunction may be one of the key mechanisms to explain the loss of gray matter observed in HAND [44].

Studies have shown that gp120 affects neuronal calcium dynamics in a number of ways, including by clustering NMDA receptors [45] and upregulating α7-nAChRs [46], leading to increased Ca^2+^ influx into neurons. Others have shown that Na + /H+ exchangers and voltage operated calcium channels contribute to gp120-induced increases in intracellular Ca^2+^ [47]. However, studies examining the effect of gp120 on ER calcium dynamics specifically are limited. ER Ca^2+^ dynamics are a major indicator of ER health, and changes in ER Ca^2+^ homeostasis leads to ER stress [48]. When this occurs, ER resident proteins are redistributed and secreted from the ER, a phenomenon that has been termed “ER exodosis” [49]. We utilized a SERCaMP assay, which relies on this phenomenon of Ca^2+^ depletion-induced ER exodosis to show that gp120 induces Ca^2+^ efflux from the ER in Gluc-ASARTDL cells. Gp120 exposure resulted in a pattern of ER calcium depletion similar to that observed with thapsigargin, though less pronounced, which was expected since thapsigargin is a potent inhibitor of the SERCA [38]. Moreover, with Ca^2+^ imaging, we found that most cells that were exposed to gp120 for 24 h either did not respond to thapsigargin or exhibited a small response, while the KCl response of these cells was unaffected, consistent with the ability of KCl to change Ca^2+^ dynamics by depolarization. These results suggest that gp120 disrupts ER Ca^2+^ dynamics by depleting ER Ca^2+^ stores, but whole cell Ca^2+^ dynamics remain functioning properly, further suggesting that gp120 induces ER stress. These findings raise the question of whether gp120 affects key ER Ca^2+^regulators, such as SERCA pumps or IP3 receptors. The underlying mechanism is complex and needs further study aimed at delineating the mechanisms by which pharmacologic modulation of ER Ca^2+^ homeostasis influences gp120-induced ER stress.

Ca^2+^ depletion from the ER is a major inducer of ER stress and contributes to the accumulation of misfolded proteins within the ER. This occurs partly because many ER chaperones that aid in protein folding are calcium dependent. Thus, when ER Ca^2+^ levels are depleted, calcium dependent chaperones lose their functionality, leading to an accumulation of misfolded proteins and subsequent ER stress and UPR activation. The activation of the UPR in response to low Ca^2+^ levels leads to the increased expression of various proteins involved in restoring cellular homeostasis. For example, the expression and secretion of certain ER proteins and chaperones, including BiP, are increased in response to ER calcium depletion. If ER calcium levels remain low, ER stress signaling will persist and these adaptive responses will become maladaptive, ultimately leading to cellular dysfunction. This includes the upregulation of pro-apoptotic factors, mitochondrial dysfunction, increased reactive oxygen species production, and caspases activation, resulting in apoptosis. This process is implicated in many diseases in which protein misfolding and aggregation contribute to neurodegeneration, such as Alzheimer’s, Parkinson’s, and Huntington’s disease [50, 51]. In fact, a number of studies have shown that HAND brains exhibit amyloid pathology [28, 52], a feature of Alzheimer’s disease. This further suggests an involvement of protein misfolding in HAND, in which ER stress may be playing a role.

**Fig. 7:**
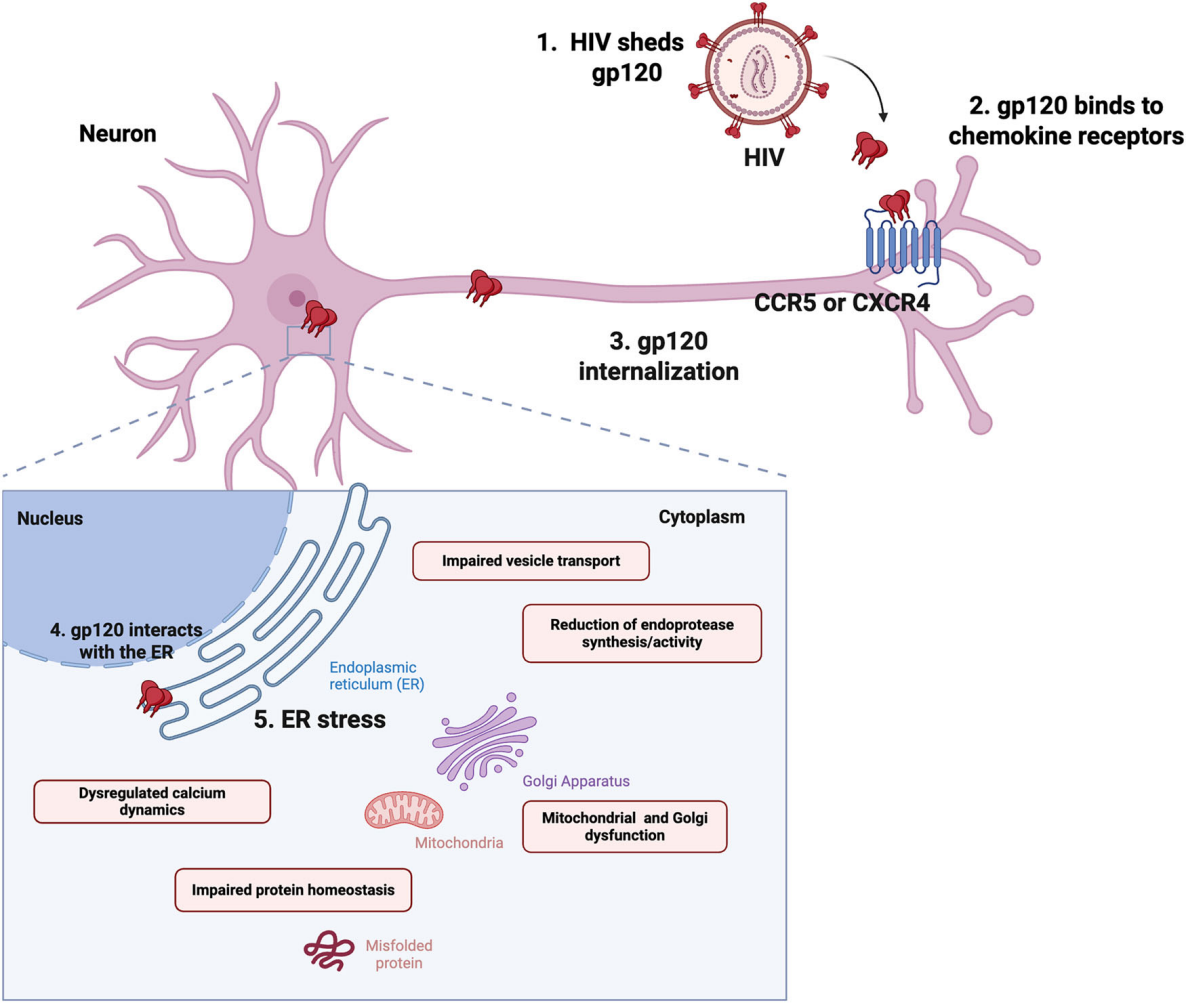
Summary of proposed mechanism. HIV enters the CNS and sheds gp120. Gp120 is internalized into neurons by binding to the chemokine co-receptors. Internalized gp120 binds to microtubules and makes its way toward the ER, where it may cause ER stress in a number of ways. These include through altered ER calcium dynamics, increased UPR activation, decreased chaperone protein function, reduced transmembrane proteins, and altered proconvertases. Gp120 may indirectly cause ER stress by affecting structures that work closely with the ER to maintain homeostasis, including mitochondria and GA. Created in BioRender. Lab, M. (2025) https://BioRender.com/4xfjvd1.

In conclusion, our data demonstrate that gp120-mediated neurotoxicity may be partly explained by gp120’s effect on ER function. The exact mechanisms by which gp120 disrupts ER homeostasis are not entirely understood and are likely influenced by multiple factors. Studies have shown that gp120 can be taken up into neurons via CXCR4 and trafficked via the mannose receptor to perinuclear compartments, including lysosomes and ER [18**–**20]. Thus, it is plausible to assume that gp120 in the ER may affect its function. Our previous data have clearly indicated that CXCR4 is necessary for initiating gp120’s neurotoxic pathway [16]. Thus, it is likely that both direct and indirect mechanisms contribute to gp120 induced ER stress. The proposed mechanisms are summarized in Fig. 7. Future studies using permeable gp120 are necessary to establish whether CXCR4 is only a carrier for gp120.

## Supplementary information

Supplementary Figure 1

Original blots

## Data Availability

The authors declare that the data supporting the findings of this study are available within the paper and its Supplementary Information files. Should any raw data files be needed in another format, they are available from the corresponding author upon reasonable request.
