# Supplementary material for: Human immunodeficiency virus 1 glycoprotein 120 induces endoplasmic reticulum stress in neurons
Source: Cell Death Dis. 2025 Oct 6;16(1):704. doi: 10.1038/s41419-025-08032-x (PMC12500905; doi:10.1038/s41419-025-08032-x)
Supplement: Supplementary file 1 — Supplementary Figure 1 [file 41419_2025_8032_MOESM1_ESM.docx]

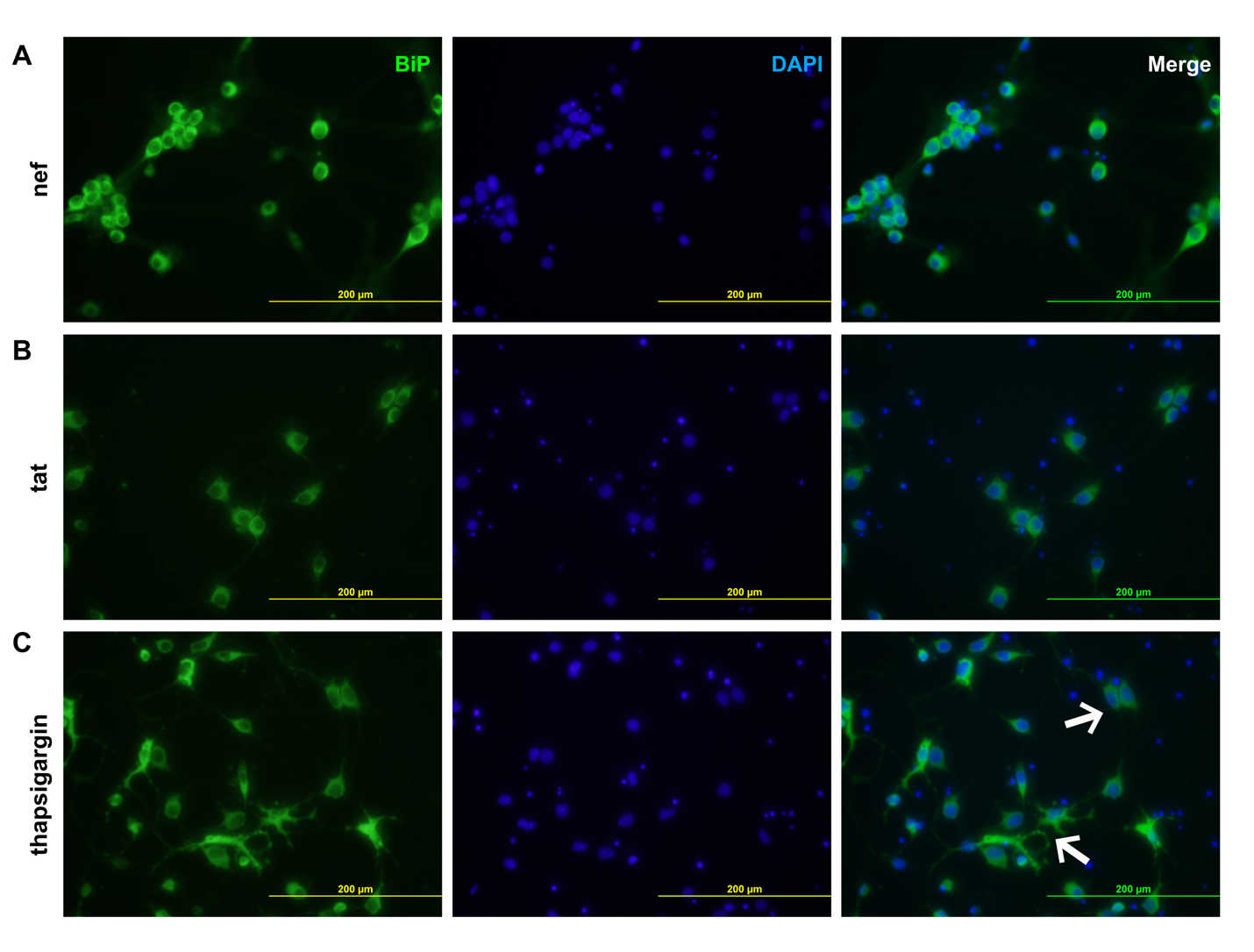


**Supplementary Figure 1.** *ER stress morphology in rat cortical neurons***.** Representative images of primary neuronal cultures. On 14 days in vitro, neurons were exposed to the HIV proteins nef **(A)** or tat **(B)**, or the SERCA inhibitor thapsigargin **(C)**. 24 hours later, cells were fixed in 4% PFA and ER morphology was observed by immunocytochemistry using an anti-BiP antibody (green). DAPI (blue) was used to stain nuclei. ER stress-associated morphology, including enlarged lumen with ER membrane, is observed in neurons exposed to thapsigargin. White arrows point to representative neurons exhibiting the described ER stress morphology. Bar = 200 μm. Images were acquired on an Olympus IX71 fluorescence microscope. The experiment was repeated three times with three different cultures of neurons (3 biological replicates). Experimental groups were randomly assigned and researchers were blinded during experimentation and analysis.
