# Supplementary figures and images for: Human immunodeficiency virus 1 glycoprotein 120 induces endoplasmic reticulum stress in neurons
Source: Cell Death Dis. 2025 Oct 6;16(1):704. doi: 10.1038/s41419-025-08032-x (PMC12500905; doi:10.1038/s41419-025-08032-x)

**Figure 1 Blots**

**BiP**

**
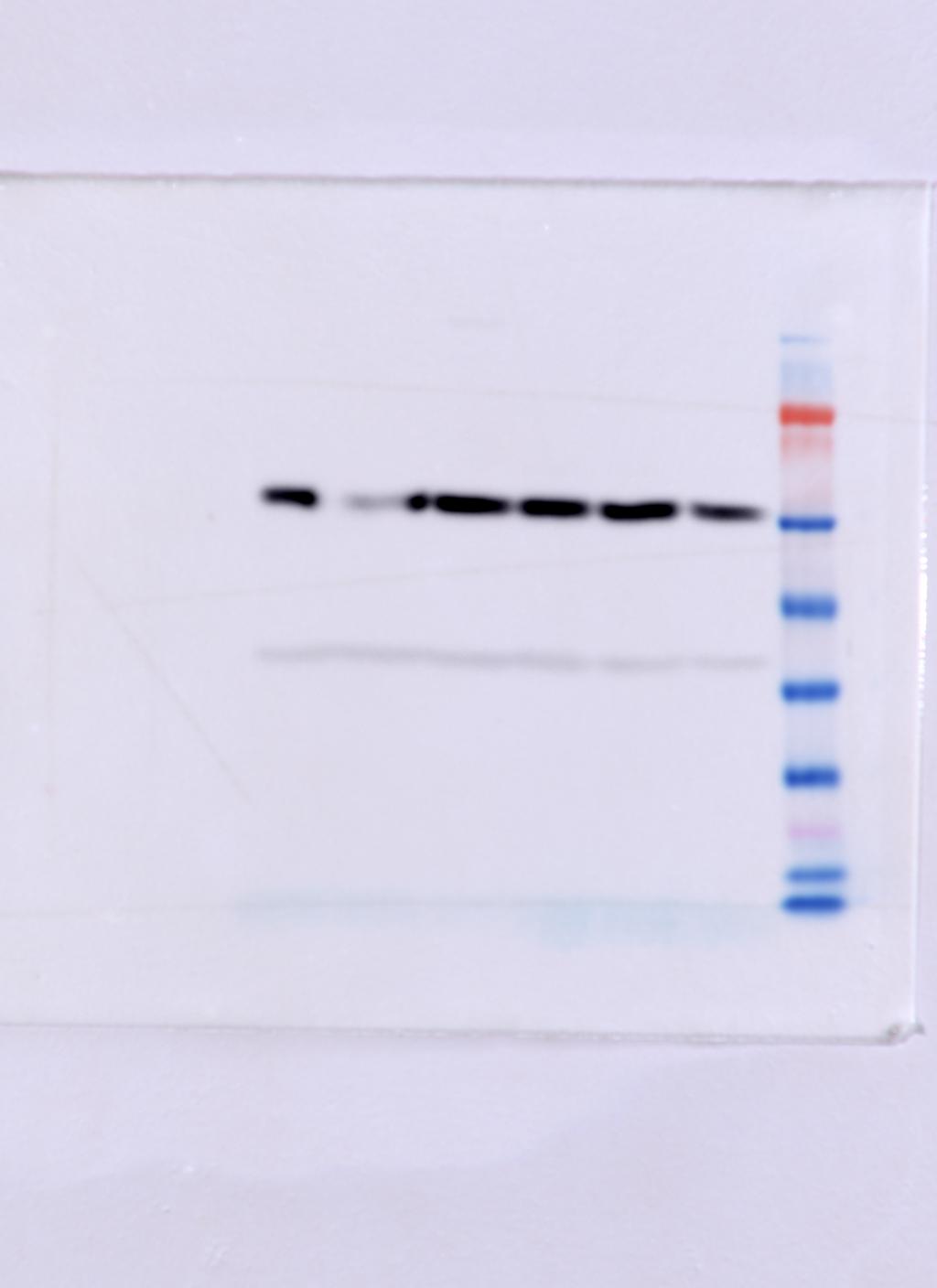
**

**p-IRE1a**

**
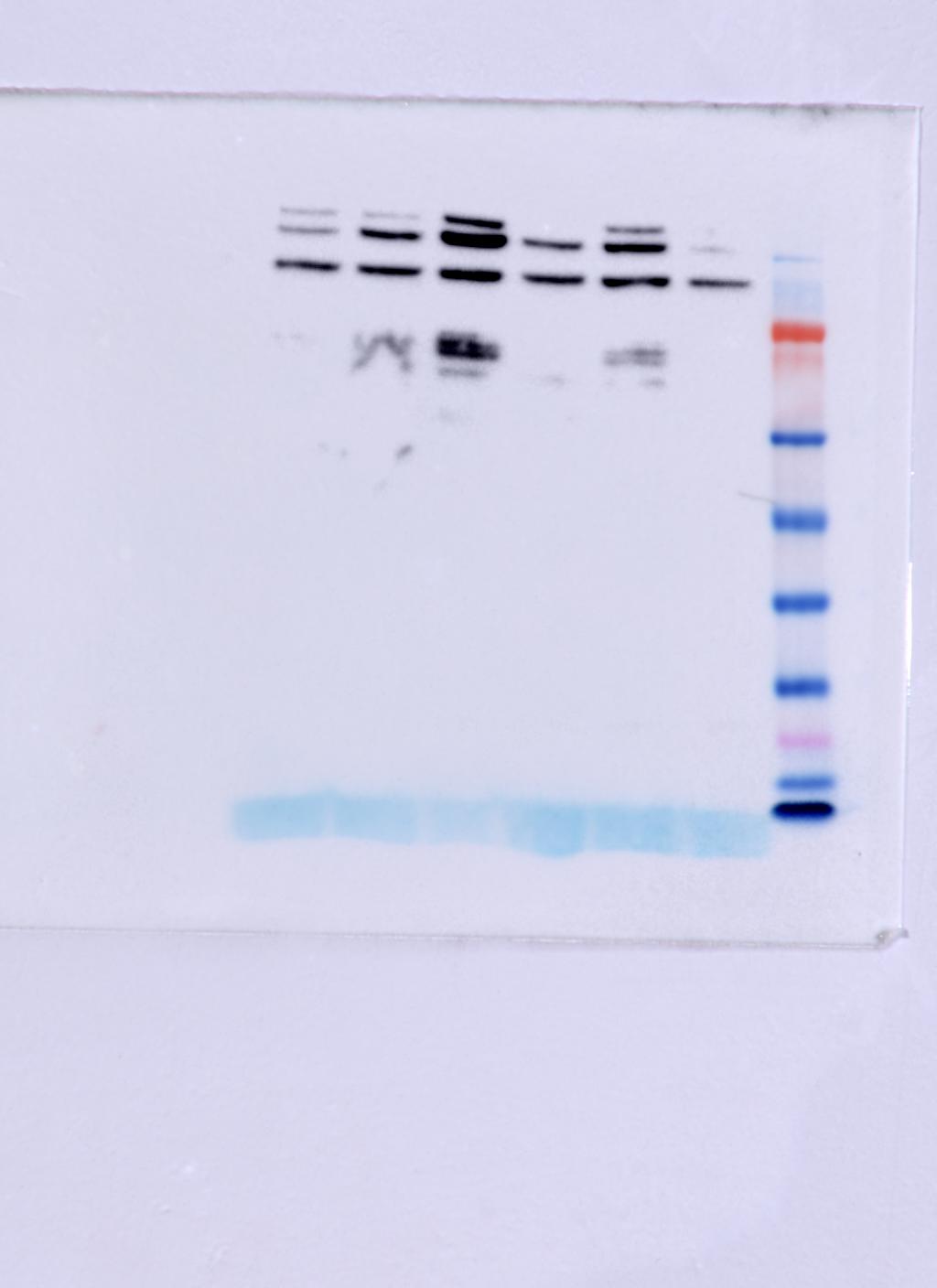
**

**CHOP**

**
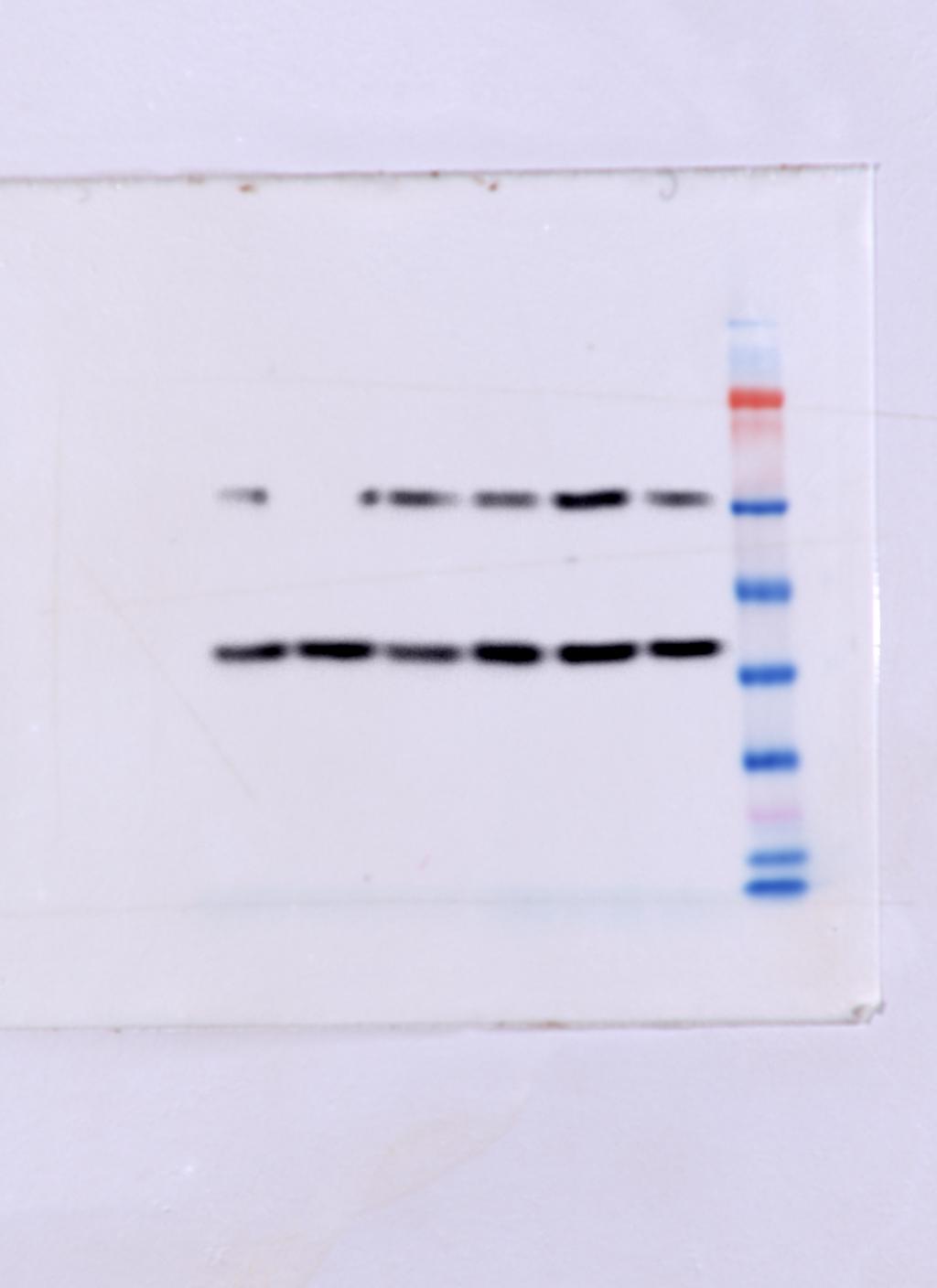
**

**Actin**

**
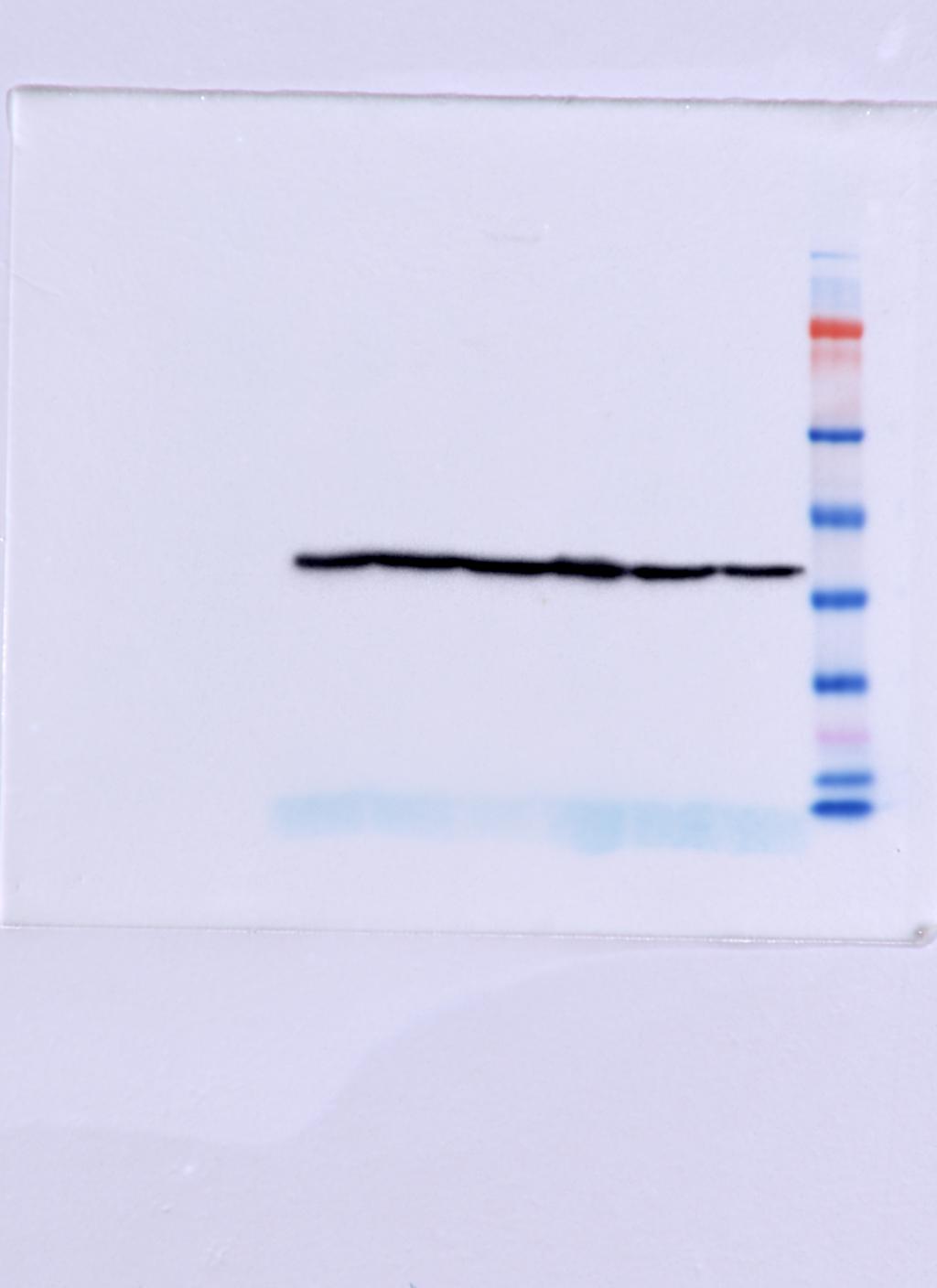
**

**Figure 3 Blots**

**BiP**

**
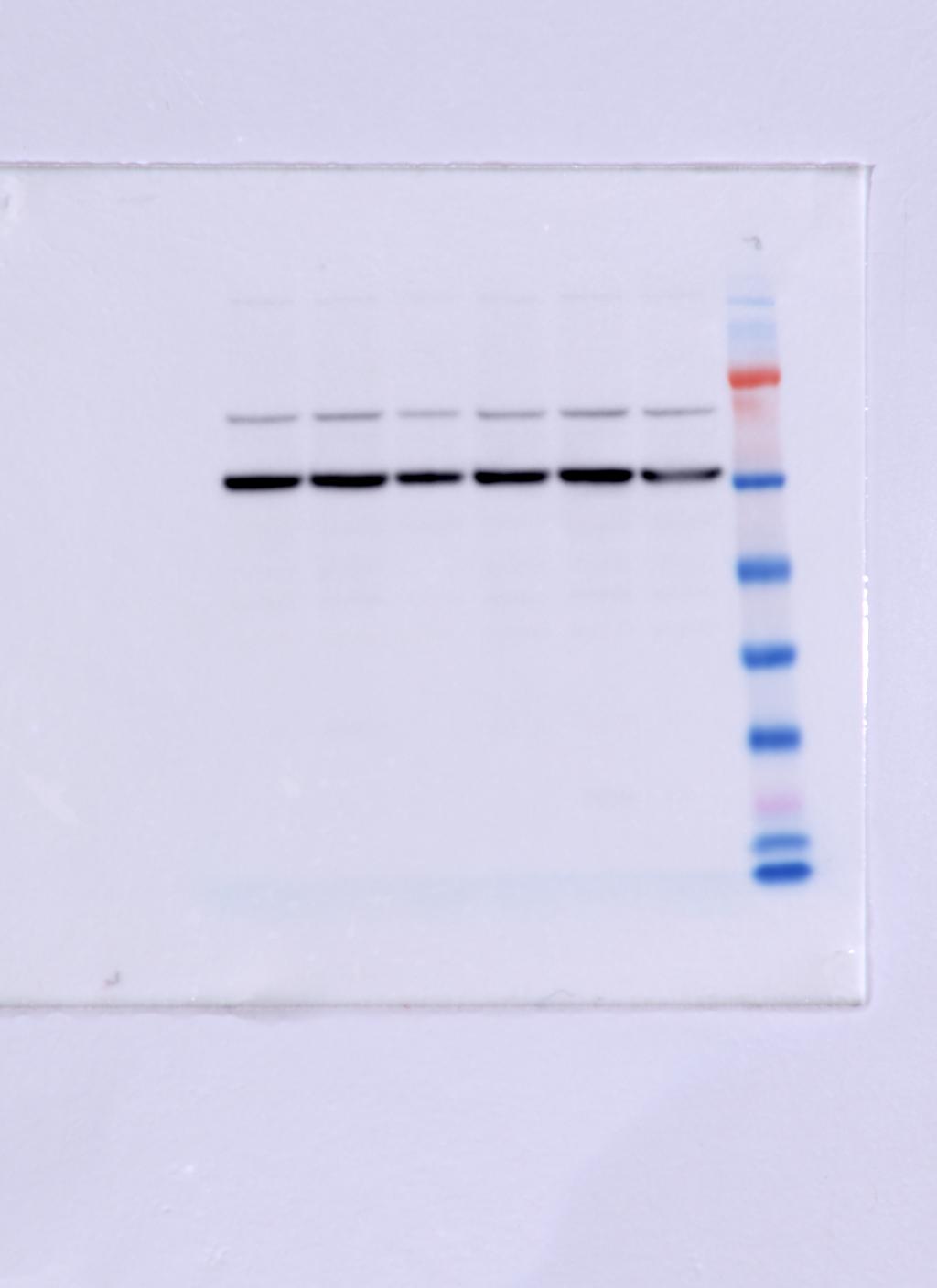
**

**p-IRE1a**

**
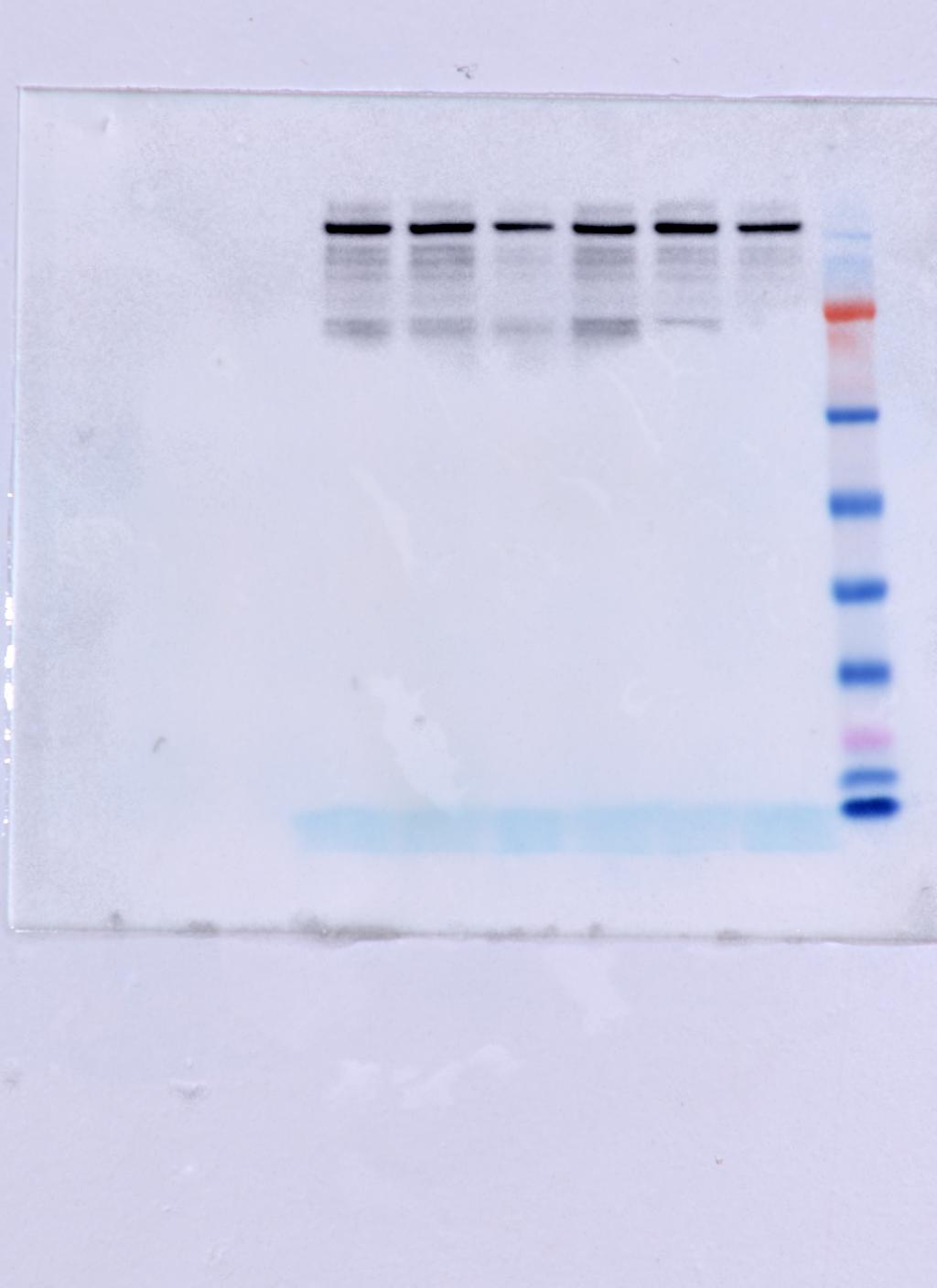
**

**CHOP**

**
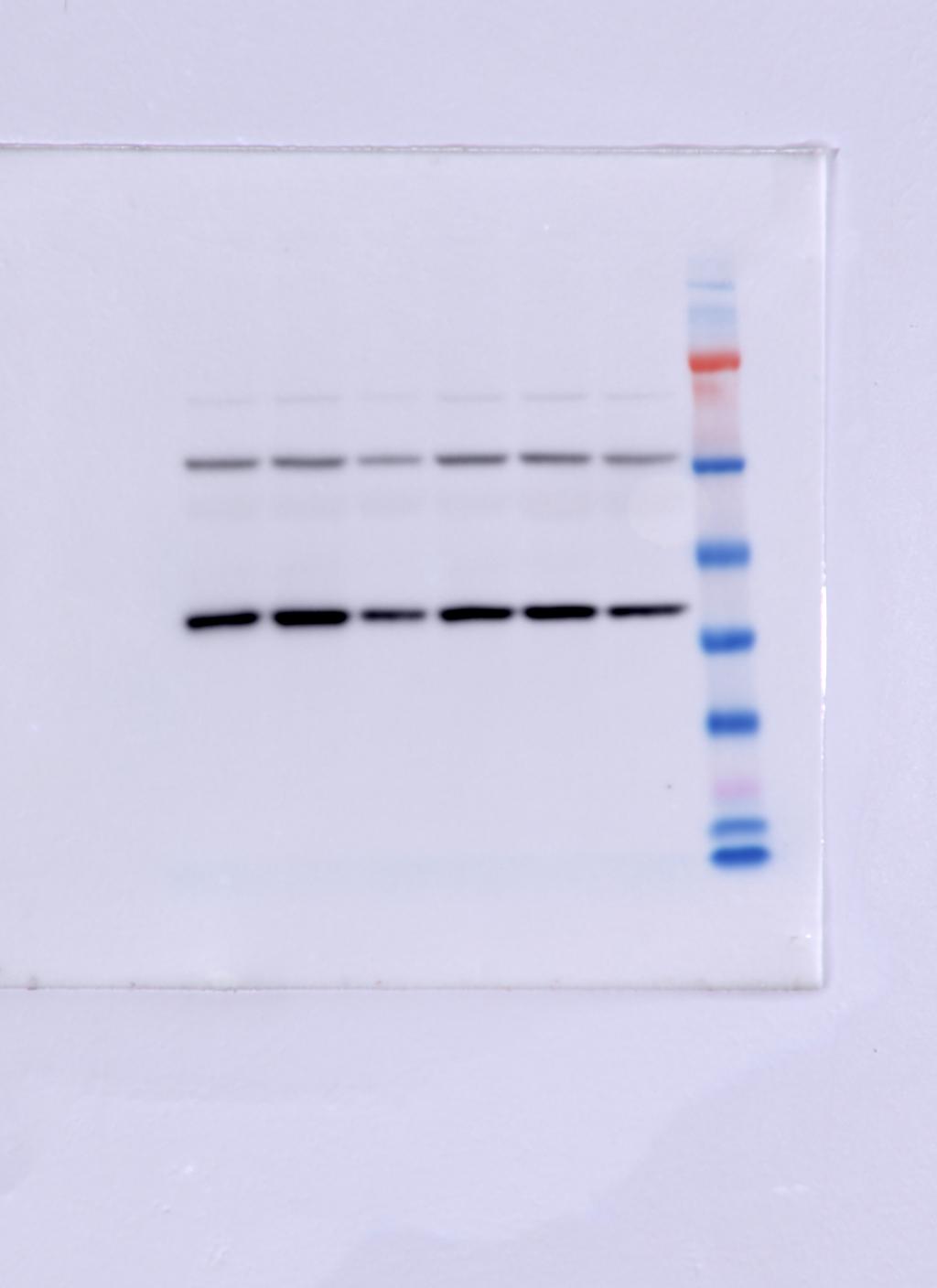
**

**Actin**


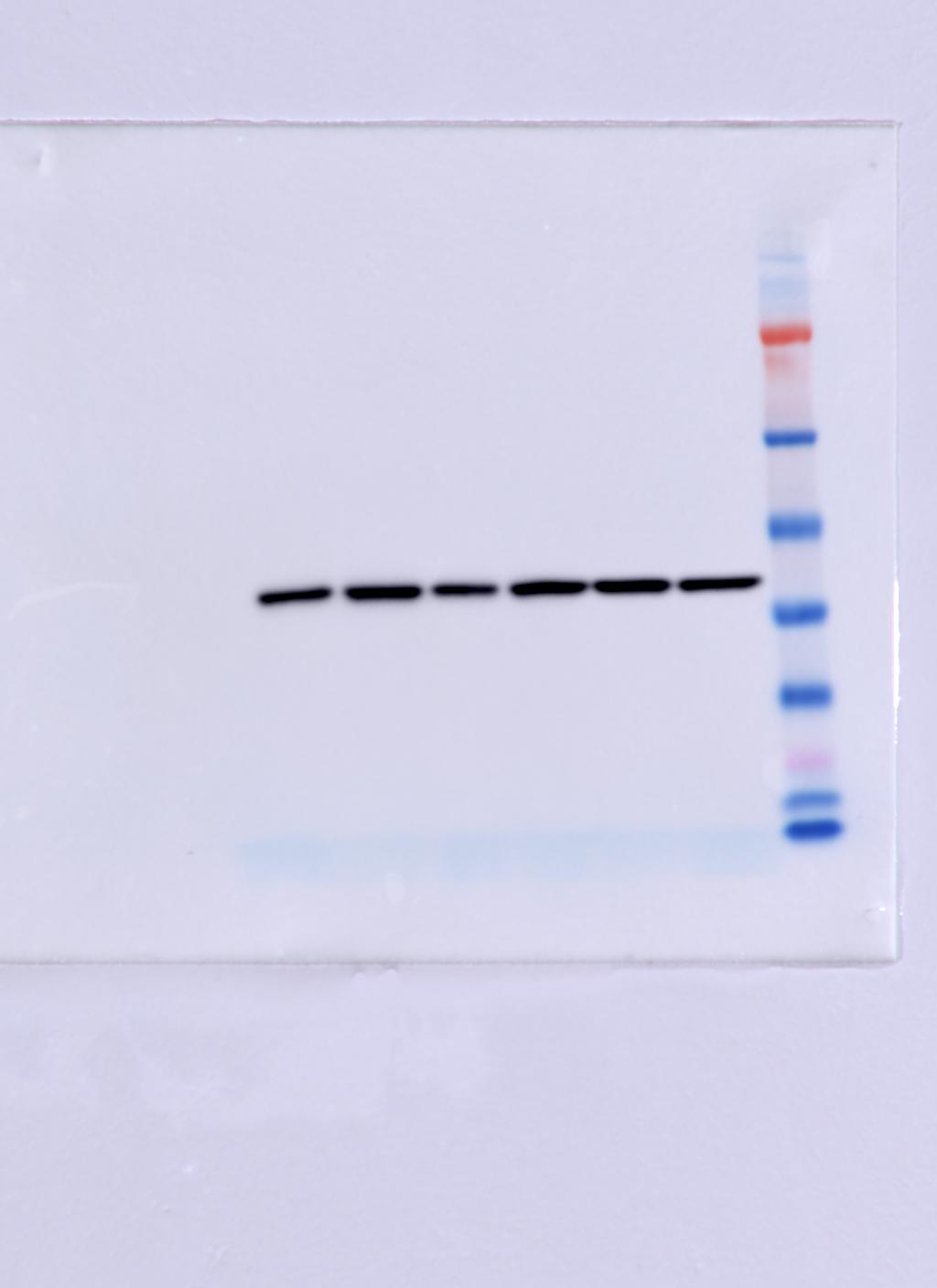

Supplement: Supplementary file 2 — Original blots [file 41419_2025_8032_MOESM2_ESM.docx]
